# Intradialytic neuromuscular electrical stimulation with optional virtual reality distraction improves not only muscle strength and functional capacity but also serum albumin level in haemodialysis patients: a pilot randomized clinical trial

**DOI:** 10.1186/s12882-023-03283-2

**Published:** 2023-08-23

**Authors:** Lena Schinner, Klaus Nagels, Julia Scherf, Christoph Schmaderer, Uwe Heemann, Claudius Küchle, Liya Hannemann

**Affiliations:** 1 Chair of Healthcare Management and Health Services Research, University of Bayreuth, Parsifalstraße 25, 95445 Bayreuth, Bavaria Germany; 2 Kuratorium Für Dialyse Und Nierentransplantation (KfH), Nierenzentrum München-Giesing, Munich, Germany; 3 Department of Nephrology, School of Medicine, Klinikum Rechts Der Isar, Technical University of Munich, Ismaninger Straße 22, 81675 Munich, Germany

**Keywords:** Neuromuscular electrical stimulation, Muscle strength, Virtual reality, Haemodialysis, Albumin, Sarcopenia

## Abstract

**Background:**

Sarcopenia is highly prevalent in haemodialysis (HD) patients and linked to a poor prognosis regarding comorbidities and premature mortality. Previous studies assessed the effects of neuromuscular electrical stimulation in haemodialysis patients. This study adds to the relevance of neuromuscular electrical stimulation (NMES) applications combined with a virtual reality (VR) distraction to increase intensity, dosage, and efficiency of NMES and slow sarcopenia progression in HD patients.

**Methods:**

We conducted a 12-week multicenter prospective randomised controlled trial. The patients were randomly assigned to one of the three groups: neuromuscular electrical stimulation with or without combined virtual reality distraction or control group.

**Results:**

The final analysis included 32 haemodialysis patients (mean age of 68 ± 10 years, 26 men). Interaction effects between groups and time (12 weeks) were significant regarding serum albumin levels (*p* = 0.008) and left quadriceps femoris muscle (QFM) force (*p* = 0.026). Both endpoints were increased in the NMES compared to the CO group at the end of the intervention. The NMES group increased serum albumin levels significantly after 12 weeks. The main effect of time was an increase in mean right QFM force between beginning and end of the intervention (*p* = 0.021). Functional capacity improved after 12 weeks in the NMES and NMES + VR but not in the control group, with a significant difference between the three groups (*p* = 0.022). Weight and body mass index increased in the NMES and NMES + VR groups, albeit not significantly. The effects of VR distraction on NMES efficiency were inconclusive.

**Conclusion:**

Intradialytic NMES increases serum albumin level, functional capacity, muscle strength in lower limb and in tendency weight and body mass index of HD patients. Effects on VR distraction are inconclusive. Large-scaled follow-up studies on integrated sports programs with NMES and active training in combination with VR as distraction and motivation accelerator are needed.

**Trial registration:**

German Clinical Trial Register: DRKS00029276 (Retrospectively registered: 30/06/2022).

## Background

As of 2019, over 850 million people worldwide suffered from a chronic renal insufficiency representing over 10% of the global population [1]. In 2010, the number of people requiring dialysis was estimated to range between 4.9–9.7 million [2]. In developed countries, increasing prevalence of arterial hypertension, diabetes mellitus, and an aging population are causing high incidences of chronic renal failure. Improving the prognosis but also the health-related quality of life of this patient group is therefore essential.

Patients on dialysis have a significantly reduced life expectancy compared to the general population. 5-year mortality ranges from 10 to over 80%, depending on age and comorbidities [3].

Sarcopenia defined as loss of skeletal muscle mass and muscle function [4] is caused by various diseases and circumstances such as aging. However, advanced renal insufficiency is also strongly associated with rapidly developing sarcopenia, and this is independent of age [5]. The prevalence of sarcopenia in dialysis patients ranges from 21–68% depending on the diagnostic algorithms applied for differential diagnosis [6, 7]. The mortality rate of sarcopenia among dialysis patients is also increased (adjusted odds ratio: 1.83 (95% CI: 1.40–2.39)) [8]. Sarcopenia implies physical limitation and increases hospitalizations due to falls and immobilization [9, 10] and is also associated with depression [11]. cardiovascular disease [12] and mobility impairments [13]. Protein-energy wasting (PEW) is also related to sarcopenia [14]. It is a maladaptive metabolic state, which can be caused by multiple factors. Some of them can be related to kidney function, others not, e. g. chronic inflammation. Among other things PEW is defined as the loss of body protein mass and energy reserves (i.e., muscle mass and muscle strength) as well as albumin level < 3.8 g/dl in patients with CKD and renal failure [15, 16]. But it should be distinguished from malnutrition as an inadequate intake of nutrients with an intact adaptive metabolic response [17]. This is because PEW or its extreme form, cachexia, is a dysfunctional condition common in inflammatory diseases and resistant to nutritional supplementation [16, 18, 19]. The prevalence is influenced due to different dialysis types, population differences, and assessment tools. Overall, a global meta-analysis found a prevalence of PEW in dialysis patients ranging from 28 to 54% [20]. An increase in serum albumin level over time is associated with better survival in haemodialysis (HD) patients [21, 22].

Due to the immobilization required for dialysis (approx. 4 h, 3 times a week) and the frequently associated comorbidities, physical activity of dialysis patients, including exercise, is markedly reduced and further decreases with time and severity of disease [23–25]. The positive effects of intradialytic training therapies, e.g. with regard to the cardiovascular health and health-related quality of life, have already been proven in randomized studies and summarized in meta-analyses [26, 27]. Because of significantly reduced general condition and limited mobility during dialysis, adherence to active training e.g., by using bicycle ergometers is either impossible or refused in these patients. Therefore, the implementation of intradialytic exercises in the clinical practice is limited [28, 29].

Neuromuscular electrical stimulation (NMES) is a form of training in which electrical impulses stimulate nerves and, thus, cause targeted muscles to contract. Hence, NMES training could improve muscle strength and functionality in everyday life in this population. However, laboratory results are inconclusive with respect to serum albumin levels and, thus, improvement of sarcopenia and PEW [30–32].

Before starting this pilot study, existing literature showed only the effects of non-immersive virtual reality (VR) training programs, like Nintendo Wii Fit and fully immersive VR programs about mindfulness for HD patients in pilot studies [33]. One limitation of NMES is the innocuous, but unpleasant tingling sensation caused by the current flowing along the limb with related impact on intensity and dose [34–36].

Fully immersive VR headsets are already being used as a distraction therapy for pain patients [37, 38]. Specific clinical evidence or guiding principles to what extent the intensity, dosage, and efficiency of NMES training can be optimized by distraction through VR glasses have not been published. Our pilot study was aimed to assess the general clinical potential of intradialytic NMES on sarcopenia, measured by muscle function and clinically relevant laboratory parameters. Additionally, we aimed to evaluate whether VR distraction was able to improve training efficiency. Based on the results of this study, a large-scale multicenter study will be designed.

## Methods

### Study design

In this randomized controlled pilot trial, we tested the effects of NMES combined with VR distraction in HD patients in the German dialysis care setting in a 12-week long intervention. In addition to muscle strength and structure indicators, we analysed body composition, functional capacity, and serum biochemistry (albumin level) as well as changes in NMES intensity due to VR distraction.

The study was approved by the Ethics Committee of the Klinikum rechts der Isar (MRI), Munich and was performed in accordance with the Declaration of Helsinki guidelines. It was also registered in the German Clinical Trial Register (DRKS00029276 | 30/06/2022). Due to low study participation rates and high dropout rates caused by uncertainties and patient deaths during the SARS-CoV-2 pandemic 2021, the study was recruited in two phases to increase the number of participants. The first 12-week study period was conducted from January 2021 at the Klinikum rechts der Isar, Munich. The second phase from April 2022 at the Kuratorium für Dialyse und Nierentransplantation (KfH) in Munich-Giesing.

### Study participants

The inclusion criteria were as follows: age of 18 years or older, haemodialysis duration more than 6 months, signed informed consent and sufficient eyesight. The exclusion criteria included cardiovascular diseases, electrical implants, metallic implants in the treatment area, susceptibility to nausea or dizziness (so-called "motion sickness"), potassium level > 7 mmol/l before intervention, acute infectious diseases (e.g., Covid-19 disease, influenza), alcohol and/or drug dependence (NMES not after alcohol consumption), increased risk of thrombosis and known dementia. Preliminary diagnoses and laboratory chemistry values were reviewed by the medical staff. Any signs of motion sickness were anamnestic and assessed by the medical staff.

### Study procedures and training protocol

The eligible patients were randomly assigned to one of the three groups: neuromuscular electrical stimulation (NMES), NMES combined with a VR distraction (NMES + VR) or control (CO) group. Randomization was generated by random.org online software [39].

**Fig. 1:**
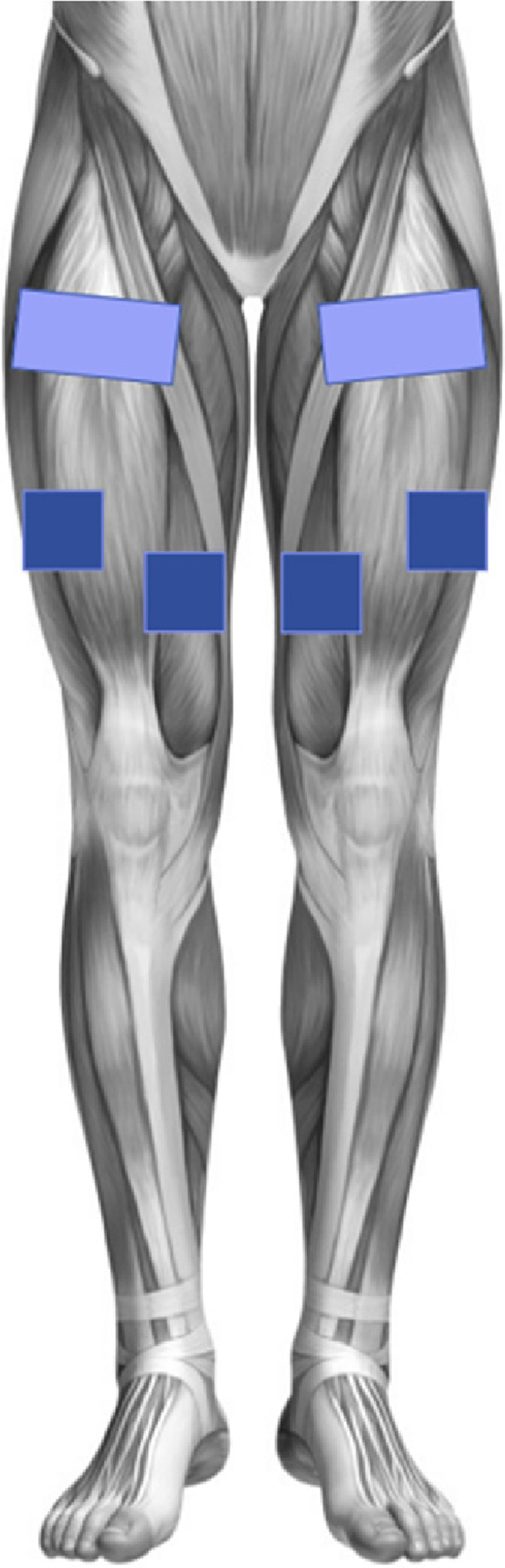
Approximate positioning of the electrodes for neuromuscular electrical stimulation of the quadriceps femoris muscle

All patients underwent the standard HD care, but patients in the NMES and NMES + VR group additionally received an intradialytic NMES of the quadriceps femoris muscles (QFM) of both lower extremities using portable 4 channel stimulation STIM-PRO X9 + (Axion, Germany). The training was applied two or three times a week, but in total 60 min a week. NMES characteristics were set up on a frequency of 20 Hz, pulse width of 250 µs and a rhythm of 5 s stimulation and 2s rest. The intensity was individually adjusted in milliampere (mA) and could be determined by the patient’s subjective sensation. Three self-adhesive reusable electrodes (One: 100 × 50 mm, two: 50 × 50 mm) were placed on each of the upper leg (Fig. 1). Patients in the NMES + VR group were distracted from the unpleasant sensation of NMES by the VR headset (Oculus Go, USA). The patients could choose between several VR applications on journey, relaxation, or interactive games. All patients were asked to continue their lifestyle as usual. The training procedures were realized by the trained study assistants and supervised by the medical staff at the MRI and KfH.

### Endpoints

#### Baseline questionnaire

The self-created baseline questionnaire included the demographic variables (age, gender, weight, duration on HD, primary disease of dialysis) and three questions on technical interest and physical condition which were rated on a 5-point Likert scale. The written questionnaire was answered by the patient at the time point before starting the interventions (t0). The baseline questionnaire was intended to minimize bias due to demographic variables and the three specific questions between groups.

#### Muscle strength

**Fig. 2:**
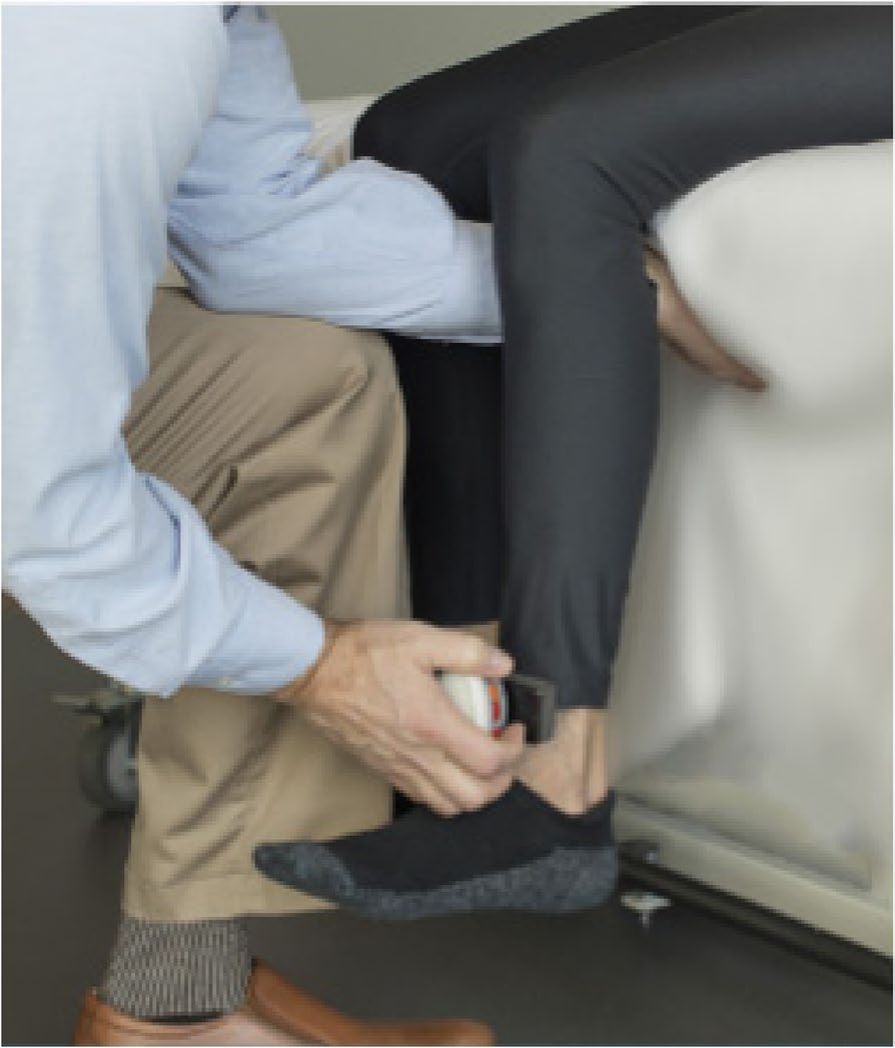
Measurement of muscle strength by using a digital handheld dynamometer microFET®2

Using a digital handheld dynamometer microFET®2 (HOGGAN SCIENTIFIC LLC, USA) the muscle strength in both lower extremities (especially quadriceps femoris muscle (QFM) left and right) was measured by isometric knee extensor strength testing. To perform the measurement, the patient sat upright on a patient couch (approx. 90° angle). The dynamometer was placed on the patient’s lower tibia, near the medial malleolus. The study assistant conducting the strength test held on to the couch to avoid losing balance due to the counterforce of the patient. The patients were asked to hold the maximum muscle effort for approximately 3 s. The patient should keep the shin as straight as possible (Fig. 2) [40]. The test was conducted at t0 and the time point after 12 weeks (t1). To avoid measurement bias, the study assistant was to be identical at t0 and t1. In addition, the measurement was performed three consecutive times and the mean was used. Due to illness, quarantine, and study staff turnover during pandemic the dynamometer tests were partially measured by different persons at t0 and t1. Therefore, there was a gross measurement error in 8 measurements (NMES: *n* = 5, NMES + VR: *n* = 2, CO: *n* = 1). These were excluded from the analysis of the muscle strength endpoints.

#### Muscle circumference

A tape measure was used to determine the location of the greatest circumference of the thigh at t0 and t1 (in centimetres).

#### Body composition

Weight, fat, water, and muscle proportion were collected using the Beurer BF 185 body scale with bioelectrical impedance analysis (Beurer, Germany) at t0 and t1 after dialysis. We tried to measure at the same day of the week. Due to illness, quarantine, and study staff turnover during pandemic we could not adhere it for every patient. Body mass index (BMI) was calculated based on weight and height.

#### Functional capacity

To measure the effects of NMES on functional capacity we decided for using the 1-Minute Sit-to-Stand Test (STS-60), because it is a reliable, valid and responsive measurement, especially if space and time are limited, as it was in our pilot study [41]. It was performed using a chair (46–48 cm high) without armrests at t0 and t1. The STS-60 consisted of movements of sitting down and standing as fast as possible without using the arms for 1 min. The patient crossed his arms in front of his body and began in seated position. The maximum number of repetitions were documented.

#### Serum biochemistry

Blood samples were drawn from the patients at t0 and t1 to measure the serum albumin level, as among others, an independent indicator for loss of muscle mass and PEW [15, 16, 42]. NMES can result in increased levels of creatine kinase (CK). Therefore, blood samples were also drawn from the patients after the first application of NMES as well as for safety reasons at t0 and t1 by the medical staff.

We did the to-/t1-measurements mostly after dialysis. If the t0-tests had to be conducted before dialysis in a few cases, then the t1-test was also conducted before dialysis. Furthermore, due to illness, quarantine, and study staff turnover during pandemic, we were unable to conduct the t0 and t1 tests at the same day, which could have had an influence on the results.

#### Virtual reality distraction – effects

The patients of the NMES and NMES + VR group rated their subjective feeling on the unpleasant tingling sensation caused by the current along the limb after every intervention on a Visual Analog Scale (VAS). It is a standardized measuring instrument for the evaluation of subjective sensations, such as pain, nausea, or similar. The VAS consists of a straight line with the lowest and highest sensation values at the ends. The patient evaluates his or her sensation in written form with a point value on the line [43]. The VAS ranged from 0 (no unpleasant feeling) to 10 (strong pain). The intensity score of NMES (in mA) was also recorded after every intervention.

### Statistical analysis

All data were reported as estimates of arithmetic mean and standard deviation except from ordinal scaled variables and those who violated normal distribution. For these we used the median and interquartile range (IQR). Fisher’s exact test was applied to explore the differences in age and primary disease between the groups. Kruskal–Wallis-Test tested the differences between the groups for the 3 baseline questions and the one-way analysis of variance for the rest of the baseline variables. The effects of the three groups (NMES, NMES + VR, control group) over time (t0 – t1) on the main endpoints was determined by a two-way repeated measures analysis of variance. The main effects of group and time were only reported if there was no significant interaction. If the interaction was significant, the one-way repeated measures analysis of variance in combination with post-hoc-Tests (Tukey-HSD) was used for each simple main effect. All requirements of the statistical tests were considered (e.g., homogeneity of variance by the Levene’s Test). The Mann–Whitney U-test was applied to test whether the intensity and the results of VAS differed between the NMES and NMES + VR groups. If the distributions of the two groups differ according to the Kolmogorov–Smirnov test, the test cannot make a statement about the difference of the medians, but of the average ranks.

Statistical analysis was performed using the IBM SPSS Statistics version 28.0. A value of *p* < 0.05 was accepted as statistically significant in all applied tests.

## Results

**Fig. 3:**
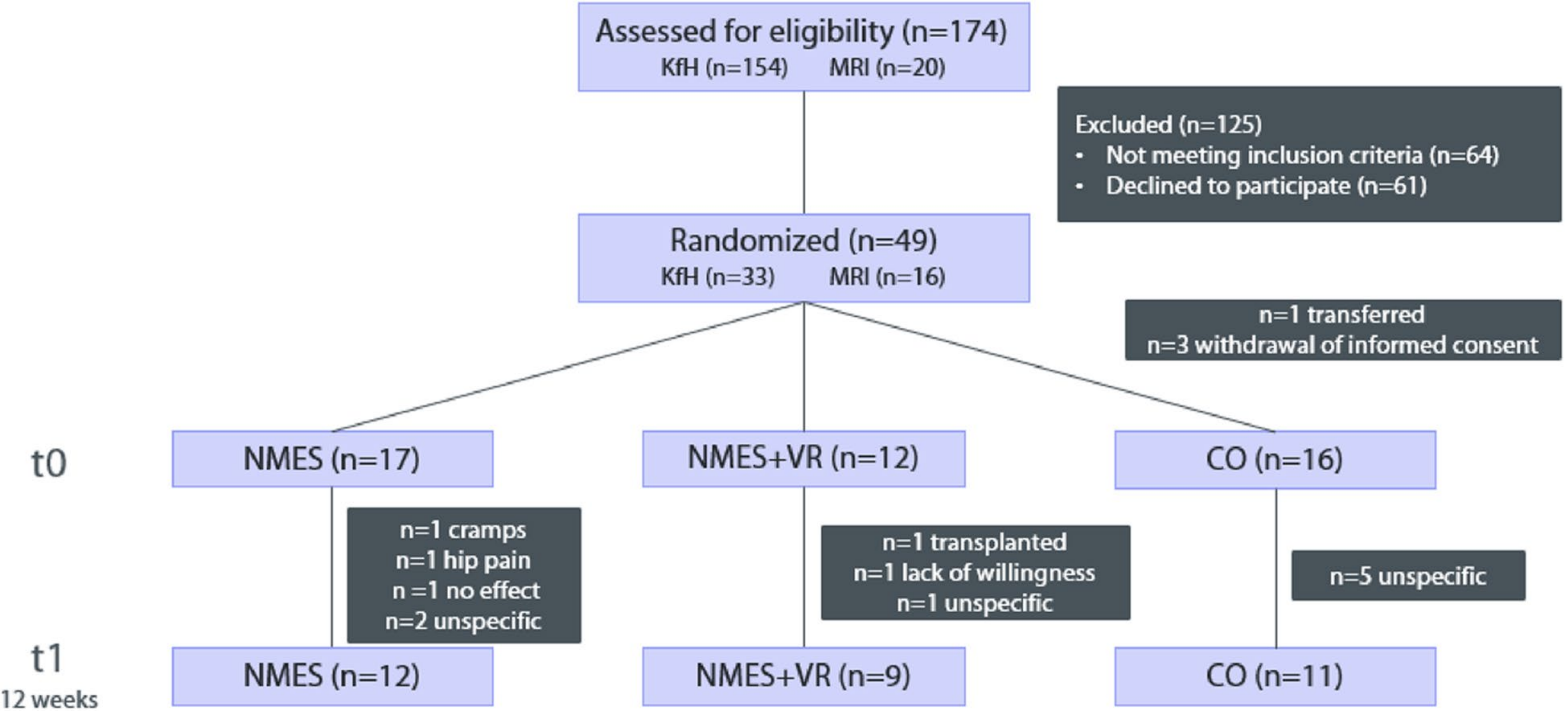
CONSORT study flow diagram. KfH: Kuratorium für Dialyse und Nierentransplantation in Munich-Giesing; MRI: Klinikum rechts der Isar; n: number; NMES: Neuromuscular electrical stimulation, NMES + VR: Neuromuscular electrical stimulation + virtual reality, CO: control, HD: haemodialysis. Note: For the endpoint muscle strength in total 8 patients (NMES: *n* = 5, NMES + VR: *n* = 2, CO: *n* = 1) were excluded because of a gross measurement error.

A total of 49 HD patients met the inclusion criteria. Before starting the first intervention four patients were excluded because of hospitalization (*n* = 1) and withdrawal of the informed consent (*n* = 3). Figure 3 gives the study flow diagram. One patient in the NMES group reported experiencing cramps three times, which caused uncertainty and led to them dropping out of the study. Another patient experienced hip pain unrelated to NMES but decided to discontinue the study. Another dropout resulted because the patient did not feel an effect due to the NMES. Most of the unspecific reasons for dropout could be due to uncertainty about COVID and a lack of willingness to continue.

The final analysis included 32 patients (12 patients in the NMES, 9 in the NMES + VR and 11 in the CO group) with a mean age of 68 ± 10 years. Most of these patients were men (81.25%). Baseline variables did not differ between groups (Table 1).

**Table 1:** Main results of the baseline variables

	***NMES group*** *n*** = *12***	***NMES* + *VR group*** *n*** = *9***	***CO group*** *n*** = *11***	***p value***
**Age (years)**	66.75 ± 9.81 67.50 (64.00–72.25)	66.22 ± 9.11 68.00 (60.50–70.50)	72.00 ± 11.39 68.00 (60.50–70.50)	0.36
**Gender (female/male in %)**	16.67/83.33	11.11/88.89	27.27/72.73	0.74
**Weight (kg)**	77.13 ± 22.54 73.75 (57.43–88.48)	71.64 ± 13.61 *68.30 (63.10–81.35)*	70.86 ± 12.68 *70.80 (62.70–76.60)*	0.65
**Body mass index (kg/m**^**2**^**)**	25.69 ± 6.90 24.45 (21.00–28.68)	24.61 ± 2.77 *24.10 (23.10–26.45)*	24.94 ± 4.08 *24.90 (21.30–29.50)*	0.88
**HD time (months)**	60.00 (14.25–93.75)	60.00 (24.00–113.50)	60.00 (30.00–96.00)	0.60
**Primary disease of HD**				0.62
*Autoimmune*	1	0	1	
*Cancer*	0	0	1	
*Diabetic nephropathy*	0	1	0	
*Hypertension*	0	1	0	
*After heart surgery*	0	0	1	
*Haemolytic, uremic syndrome*	1	0	0	
*Polycystic kidney disease*	1	2	1	
*Due to genetics*	0	1	0	
*Unknown*	9	4	7	
**Baseline questions**				
*Q1: technical interest*	3 (3–4)	4 (1.5–4.5)	4 (2–5)	0.94
*Q2: physical status*	3 (2–3.8)	3 (2–3.5)	3 (1–5)	0.96
*Q3: disability due to HD*	3 (2–3)	4 (2–4)	3 (2–4)	0.64

Results expressed as mean (standard deviation) and/or median (interquartile range)

Q1: I am interested in technology and in this respect, I like to be up to date with the latest technology.; Q2: My physical condition affects my everyday life, e.g., out of fear of falling.; Q3: Haemodialysis makes me feel restricted in my physical activity.; Q1-Q3 rated by a 5-likert scale

*NMES* Neuromuscular electrical stimulation, *NMES + VR* Neuromuscular electrical stimulation + virtual reality, *CO* control, *HD* haemodialysis; Statistical significance: *p < 0.05 comparison between groups

Table 2 gives all results of two-way repeated measures analysis of variancefor the endpoints.

**Table 2:** Changes in functional capacity, serum biochemistry (albumin level), muscle strength, muscle circumference and body composition

		***n***	***Baseline (t0)***	***12 weeks (t1)***	Time* (p value)	Repeated measures ANOVA* (p value)
**Functional capacity**	**STS-60 (n)**					**Group 0.022** **Time 0.962** **Interaction 0.447**
	NMES	11	27.82 ± 9.49 29.50 (19.25–32.00)	28.09 ± 14.31 27.00 (21.00–32.00)		
			**0.97%**			
	NMES + VR	9	20.33 ± 5.59 22.00 (14.50–25.00)	22.89 ± 6.94 26.00 (14.50–27.50)		
			**12.59%**			
	control	11	18.00 ± 9.24 16.00 (13.00–27.00)	15.40 ± 9.28 18.00 (8.25–19.75)		
			**-14.44%**			
**Serum biochemistry**	**Serum albumin level (g/dl)**					**Interaction 0.008**
	NMES	12	4.10 ± 0.26 4.10 (4.00–4.35)	4.33 ± 0.45 4.35 (4.025–4.48)	0.030	
			**5.61%**			
	NMES + VR	9	3.99 ± 0.34 4.10 (3.65–4.30)	4.08 ± 0.35 4.00 (3.75–4.40)	0.347	
			**2.26%**			
	control	11	4.04 ± 0.40 4.10 (3.80–4.20)	3.71 ± 0.71 3.90 (3.60–4.00)	0.081	
			**-8.17%**			
	Group* (p value)		0.747	0.032		
**Muscle strength**	**QFM force right (kgf)**					**Group 0.807** **Time 0.021** **Interaction 0.082**
	NMES	7	13,30 ± 5,65 12.70 (9.10–19.80)	17,77 ± 3,27 18.30 (15.30–19.90)		
			**33.61%**			
	NMES + VR	7	14.05 ± 4.31 15.70 (10.30–16.66)	15.21 ± 6.09 14.20 (10.50–18.40)		
			**8.26%**			
	control	10	12.81 ± 3.75 10.90 (9.64–16.80)	12.98 ± 5.09 12.45 (9.53–17.18)		
			**1.33%**			
	**QFM force left (kgf)**					**Interaction 0.026**
	NMES	7	14.26 ± 5.20 15.20 (10.10–17.86)	17.91 ± 3.61 17.80 (15.20–20.60)	0.059	
			**25.60%**			
	NMES + VR	7	15.75 ± 5.86 16.76 (11.70–18.73)	13.74 ± 4.71 13.10 (9.10–19.10)	0.130	
			**-12.76%**			
	control	10	11.07 ± 3.96 9.77 (8.09–15.85)	13.08 ± 3.19 13.95 (10.70–15.20)	0.132	
			**18.16%**			
	Group* (p value)		0.157	0.044		
**Muscle acircumference**	**QFM circumference right (cm)**					**Group 0.402** **Time 0.090** **Interaction 0.534**
	NMES	12	46.67 ± 7.45 44.50 (41.50–50.25)	44.54 ± 7.35 45.00 (39.13–46.00)		
			**-4.56%**			
	NMES + VR	9	42.72 ± 3.87 42.00 (38.75–46.00)	42.39 ± 3.66 43.00 (39.25–45.50)		
			**-0.77%**			
	control	11	43.68 ± 4.83 45.00 (38.00–47.00)	42.68 ± 5.34 44.00 (38.00–47.00)		
			**-2.29%**			
	**QFM circumference left (cm)**					**Group 0.281** **Time 0.291** **Interaction 0.157**
	NMES	12	46.33 ± 6.29 46.00 (42.50–48.75)	44.88 ± 7.21 45.50 (38.88–47.00)		
			**-3.13%**			
	NMES + VR	9	42.06 ± 2.86 42.00 (39.00–44.50)	42.89 ± 3.68 44.00 (39.00–45.75)		
			**1.97%**			
	control	11	43.18 ± 4.21 43.00 (39.50–47.00)	42.23 ± 5.26 44.00 (39.00–45.00)		
			**-2.20%**			
**Body composition**	**Weight (kg)**					**Group 0.586** **Time 0.684** **Interaction 0.146**
	NMES	12	77.13 ± 22.54 73.75 (57.43–88.48)	77.66 ± 22.78 73.05 (58.73–89.33)		
			**0.69%**			
	NMES + VR	9	71.64 ± 13.61 68.30 (63.10–81.35)	71.81 ± 13.41 *68.60 (63.20–80.95)*		
			**0.24%**			
	control	11	70.86 ± 12.68 70.80 (62.70–76.60)	69.70 ± 12.24 71.10 (60.90–76.00)		
			**-1.64%**			
	**BMI (kg/m**^**2**^**)**					**Group 0.160** **Time 0.390** **Interaction—(violation of equality test of covariance matrices)**
	NMES	12	25.69 ± 6.9 24.45 (21.00–28.68)	25.78 ± 6.93 24.30 (21.35–28.95)		
			**0.35%**			
	NMES + VR	9	24.61 ± 2.77 *24.10 (23.10–26.45)*	24.63 ± 2.81 *24.10 (22.90–26.45)*		
			**0.08%**			
	control	11	24.94 ± 4.08 *24.90 (21.30–29.50)*	24.60 ± 3.78 *24.90 (21.07–28.00)*		
			**-1.36%**			
	**Fat proportion (%)**					**Group 0.821** **Time 0.849** **Interaction 0.373**
	NMES	7	31.54 ± 6.14 *31.70 (24.45–36.40)*	27.01 ± 7.32 *29.30 (20.70–32.15)*		
			**-14.36%**			
	NMES + VR	6	29.28 ± 4.54 *28.30 (27.40–34.00)*	33.65 ± 10.54 *31.25 (27.78–38.23)*		
			**14.92%**			
	control	9	29.49 ± 5.12 *30.50 (25.70–31.90)*	31.10 ± 12.86 *33.20 (17.65–39.80)*		
			**5.46%**			
	**Muscle proportion (%)**					**No ANOVA no variance of homogeneity (If the variances are unequal. this can affect the Type I error rate.)**
	NMES	7	34.50 ± 3.34 *33.80 (31.40–37.30)*	37.93 ± 2.35 *38.20 (37.35–39.05)*		
			**9.94%**			
	NMES + VR	6	34.37 ± 1.42 *34.70 (34.20–35.00)*	34.15 ± 4.87 *36.40 (28.58–37.45)*		
			**-0.64%**			
	control	9	34.14 ± 2.17 *33.95 (31.73–35.43)*	32.61 ± 6.87 *28.10 (27.20–41.05)*		
			**-4.48%**			
	**Water proportion (%)**					**Group 0.821** **Time 0.966** **Interaction 0.312**
	NMES	7	50.59 ± 4.55 *50.50 (46.95–55.85)*	53.94 ± 5.40 *52.30 (50.15–58.60)*		
			**6.62%**			
	NMES + VR	6	52.27 ± 3.34 *53.00 (48.50–53.70)*	49.90 ± 5.73 *50.75 (47.00–53.33)*		
			**-4.53%**			
	control	9	53.17 ± 3.77 *52.75 (51.05–55.40)*	51.98 ± 8.73 *53.60 (42.95–60.85)*		
			**-2.24%**			

Results expressed as mean (standard deviation), median (interquartile range)and differences of means from t0 to t1 in %

ANOVA: analysis of variance: t0: baseline; t1: after 12 weeks; QFM: Quadriceps femoris muscle; BMI: Body mass index; STS-60: 1-Minute Sit-to-Stand Test; NMES: Neuromuscular electrical stimulation; NMES + VR: Neuromuscular electrical stimulation + virtual reality; CO: control; Statistical significance: *p < 0.05

^*^The two-way repeated measures analysis of variance was the major method used. The main effects of group and time were only reported if there was no significant interaction. If the interaction was significant. the one-way repeated measures analysis of variance was used for each simple main effect

### Functional capacity

The main effect of the NMES intervention was a statistically significant difference in the improvement of physical strength. This was evident in the 1-Minute Sit-to-Stand Test. While patients in the NMES and NMES + VR groups increased their numbers of repetitions, the number decreased in the CO group (Table 2).

### Serum biochemistry

There was an increase in serum albumin levels in the NMES intervention groups from t0 to t1, while we observed none in the control group. For the NMES group the increase in serum albumin levels were significantly (0.233 ± 0.094 g/dL, *p* = 0.030) (Table 2).

### Muscle strength

There was a statistically significant interaction between the intervention and time on left QFM force (F(2,21) = 4.24, *p* = 0.026, partial η^2^ = 0.292). Therefore, simple main effects analysis was run. Left QFM force was statistically significantly greater in the NMES compared to the CO group at t1 (4.83 ± 1.87 kgf, *p* = 0.044). The main effect of time differed significantly in mean right QFM force at the different time points, F(1,21) = 6.207, *p* = 0.021, partial η^2^ = 0.228 (Table 2).

### Muscle cicumference

We observed no difference in the development of muscle circumference. The circumference tended to decrease in left as well as right QFM in all groups, except from QFM left in the NMES + VR group (t0: 42.06 ± 2.86 cm vs. t1: 42.89 ± 3.86 cm) (Table 2).

### Body composition

Body composition did not change throughout our study. To avoid a Type I error rate due to no homogeneity of variance, two-way repeated measures analysis of variance was not run for the endpoint muscle proportion and interaction effect was not interpreted for the endpoint BMI due to violation of equality test of covariance matrices. Weight and BMI increased in the NMES and NMES + VR groups but decreased in the CO group. Fat proportion decreased in the NMES group by 14.36% from t0 to t1 and increased in the NMES + VR group by 14.92% and CO group by 5.46%. The same tendencies according to the changes from t0 to t1 were observed in water proportion. Muscle proportion was increased in NMES group by 9.94% from t0 to t1 and decreased in the NMES + VR and CO group (Table 2). However, the measurements of fat, water and muscle proportion were limited due to 10 erroneous measurements caused e.g., by foot deformities and limited electrical conductivities.

### Effects of VR distraction in combination with NMES

**Fig. 4:**
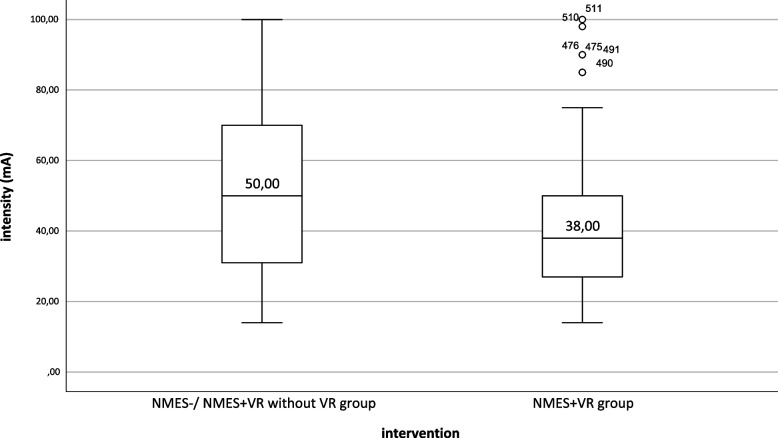
Boxplot—Individual intensity (milliampere) of stimulation in each group. Adverse events. mA: milliampere; NMES: Neuromuscular electrical stimulation; NMES + VR: Neuromuscular electrical stimulation + virtual reality. Note: For the evaluation of the VAS, 429 interventions of the NMES group and 98 of the NMES + VR group could be considered in the analysis

In 98 of the 228 interventions the patients in the NMES + VR group used to wear the VR-headsets, which means an adherece rate of 42.98%. 70.41% of the applications were of the category relaxation, 9.18% of the category interactive games and for 20.41% there was no documentation of the chosen VR applications. There were different reasons for the non-willingness to use the VR-headset. Some patients preferred to watch TV, others were bored with the applications, and others were generally not interested in the VR headset anymore. Nevertheless, the NMES was always performed, even without VR. The values of the VAS and intensity of NMES of the NMES + VR group without VR were assigned to the NMES group accordingly. Therefore, the analysis of the VAS and intensity resulted in two different numbers of interventions in the NMES and NMES + VR groups. A Mann–Whitney U test was applied to test whether the intensity of NMES differed between the NMES and NMES + VR groups. According to the Kolmogorov–Smirnov test, the distributions of the two groups differ from each other (*p* = 0.002). Therefore, only a statement about the Mean Rank can be made. There was a significant difference between the NMES (Mean Rank = 275.45) and NMES + VR group (Mean Rank = 213.88) (U = 16,109.00, Z = -3.616, *p* < 0.001). Figure 4 shows the boxplot with the median of both groups. For the evaluation of the VAS, 428 interventions of the NMES group and 98 of the NMES + VR group could be considered in the analysis. The Kolmogrorov-Smirnov test resulted in no distinction of the group distributions (*p* = 0.650), allowing the test to identify a statement about the difference in medians. The two groups NMES- (4 (IQR: 2–5)) and NMES + VR-group (4 (IQR: 0–6)) did not differ significantly from each other (U = 20,335.00, Z = -0.476, *p* = 0.634). The median VAS score was 4 in both NMES groups, which means the feeling of "tingling".

One patient, who was included in the final analysis, reported having a one-time cramping feeling in the hip area after NMES. The NMES intensity was subsequently reduced, and no further problems were reported.

## Discussion

Sarcopenia in dialysis patients is associated with physical limitations, hospitalizations, and depression. Thus, its onset and progression should be either fully prevented or at least slowed down. This randomized controlled trial design was used to evaluate the effects of intradialytic NMES combined with VR distraction in HD patients over 12 weeks. As far as we know, this study is the first to extend NMES with VR distraction in HD patients and the first in a common German dialysis care setting on this topic.

In this study, NMES significantly increased muscle strength and serum albumin levels.

Consistent with our positive results on functional capacity measured by the increased maximum number of repetitions in both NMES groups during STS-60, other studies also reported an improvement in time needed to perform 10 repetitions Sit-to-Stand Test [34, 44] and maximum number of repetitions during 30 s [45]. However, meta-analysis on the three studies was associated with considerable uncertainty (best estimate NMES improved results on the Sit-to-Stand test with a standardised mean difference of 0.42 (95% CI -0.04 to 0.87)) [30]. Most of the previous studies used the 6-min walk distance [34, 44–48]. The distance improved by a mean of 31 m (95% CI 13 to 49) [30]. We decided for the STS-60 for efficiency reasons. A systematic review summarized literature to the STS-60 and concluded that it is a reliable, valid, and responsive measurement, especially if the space and time are limited [41]. NMES seems to be an effective training to improve functional capacity and could thus reduce functional impairments in daily life.

Albumin levels increased in both NMES groups from t0 to t1 while they decreased in the CO group. These finding are not consistent with previous research [34, 44, 47–50], which were summarized by a meta-analysis and resulted in no clear effects on albumin (MD -0.04, 95% CI -0.15 to 0.08, I^2^ = 0, Q = 2.796) [30]. As serum albumin levels are a parameter to determine the sarcopenia status as well as malnutrition, we hypothesize that NMES has a positive effect on serum albumin levels in HD patients and, thus, could impact protein energy wasting and sarcopenia [5, 14–16, 42, 51].

In our study, right QFM force increased significantly. The strongest increase was observed in the NMES group followed by NMES + VR and controls. These beneficial effects of NMES on muscle strength in HD-patients are consistent with previous studies [30, 31]. Although we also found a significant interaction effect on the left QFM force in our study, the descriptive analyses showed inconclusive results, especially the reduction of muscle force in the NMES + VR group (t0: 15.75 ± 5.86 kgf vs. t1: 13.74 ± 4.71). The statistical inconclusiveness of Mqf force is probably due to the exclusion of 8 measurements due to the gross measurement error described above.

The circumference of the lower limb was assessed. A tendency to decrease was observed in all groups from t0 to t1, except from left QFM in the NMES + VR group (t0: 42.06 2.086 cm vs. t1: 42.89 ± 3.86 cm).

Previous studies used different methods to assess the muscle architecture such as, magnetic resonance imaging [49], ultrasound [45] and analysis limb circumference and skinfold thickness [34, 44]. Beneficial effects with NMES over the CO group could only be identified by Suzuki et al. by measuring the total muscle cross-sectional area with magnetic resonance imaging [49]. Due to the smaller cross-sectional area of contractile tissue in dialysis patients compared to healthy individuals [30, 49, 52] accurate methods such as MRI seem to be more suitable for further studies related to NMES and HD patients, as Valenzuela et al. have previously noted [30].

NMES had no effect on body composition, but weight and BMI tended to increase in NMES and NMES + VR.

A low BMI in dialysis patients is associated with malnutrition and a catabolic state. Therefore, it is an independent predictive marker for higher mortality, rendering an increasing weight beneficial [51]. The numbers in percentage of fat, water and muscles were incomplete caused by foot deformities and limited electrical conductivities. Simó et al. measured body composition also by the electrical bioimpedance analysis using another body scale and reported no missing data [44]. Nevertheless, this method of measuring body composition has been controversial since measurement differences could result depending on the particular impedance analysers [53]. In our pilot study we decided to use a mobile body scale with bioimpedance analysis which could be easily transported to the respective dialysis centre. For the large-scale follow-up study the measurement method and the quality of the bioimpedance analysis scale needs to be revaluated.

Only one patient reported having a cramping feeling. Therefore, NMES and NMES + VR were not associated with serious adverse events in this study. In contrast to most of the previous studies, our study resulted in a higher dropout rate of 35% (vs. < 15%) [34, 44, 45, 47–50]. We see this increased drop-out rate most likely caused by the SARS-CoV-2 pandemic, which lead to uncertainties, fears [54, 55] and dropouts due to Covid-19 disease, especially during our phase 1 in 2021.

The effects of the VR distraction regarding the tingling NMES sensation measured by the VAS score and the acceptance of higher intensity levels in the NMES + VR group were inconclusive. All the patients in the NMES + VR group seemed to be bored after some application and therefore did not use the VR headset anymore. In addition to an expanded range of programs, VR applications could be individualized for HD patients (f. e. applications to improve cognitive skills or health literacy in the context of nutrition and activity). VR applications in the context of an intradialytic physical exercise program seem to be effective and feasible [56–58]. Therefore, in the context of an integrated intradialytic sports program, VR could also serve as motivation accelerator for HD patients who can do physical exercise. Extensions in the sense of Meta Quest (successor of Oculus Go), could create interactions and entertainment through additional Metaverse-feature.

Although we showed beneficial results of NMES in HD patients, the results of this pilot study so far, are limited due to the relatively small number of patients and short study duration. Furthermore, the study was complemented by a second study phase in 2022 due to low participation and high dropout rates caused by uncertainties and deaths among patients during the 2021 SARS-CoV-2 pandemic in phase 1. The study assistants failed to adhere to the instruction to use the same measurement person for the dynamometer tests at t0 and t1 due to illness and staff changes. Thus, a gross measurement error resulted in 8 cases. Most of the patients of NMES + VR group did not apply the VR headset during every intervention of the 12-weeks. Finally, a selection bias should be considered due to the informed consent as well as the inclusion and exclusion criteria.

The follow-up study should involve a larger sample size and a longer study period to determine long term effects. Regarding the used devices, the body composition measurements should be performed by a higher quality electric bioimpedance analysis scale. Also, errors in dynamometer measurement must be consistently avoided in follow-up studies. As already suggested from Suzuki et a al. [49], further studies should compare the effects on NMES associated with other interventions. Although Dobsak et al. [48] have previously conducted a study on NMES associated with bicycle ergometer and control, this was limited by a small number of patients. VR components could also be added to a larger and methodologically rigorous follow-up study, not only for distraction but also for motivation. The overall goal for future studies of intradialytic trainings should include the option of active training, e.g. on a cycle ergometer, or passive training with NMES, depending on general condition. In fact, not only the training methods have to be investigated, but also human and cost resources in terms of implementation need to be examined so that intradialytic training can be integrated into the daily dialysis routine to maintain muscle strength and function from the onset of dialysis and prevent sarcopenia.

## Conclusions

The study results suggest that intradialytic NMES increases (1) functional capacity, (2) serum albumin level, (3) muscle strength in lower limb and (4) weight and BMI of HD patients. (5) Effects on VR distraction are inconclusive and must be proven in further studies. VR applications must be further optimized and expanded. (6) Large-scale follow-up studies on integrated sports programs with NMES and active training in combination with VR as distraction and motivation accelerator are needed. Cost-effectiveness should be evaluated to successfully implement integrated exercise programs in daily dialysis care. However, results strongly suggest to further assess the clinical potential of the combined NMES and VR interventions in HD patients.

## Data Availability

The datasets used and/or analysed during the current study are available from the corresponding author on reasonable request.

## Abbreviations

BMI: Body mass index
CK: Creatine kinase
CO: Control
HD: Haemodialysis
IQR: Interquartile range
KfH: Kuratorium für Dialyse und Nierentransplantation
mA: Milliampere
MRI: Klinikum rechts der Isar
NMES: Neuromuscular electrical stimulation
NMES + VR: Neuromuscular electrical stimulation + virtual reality
PEW: Protein-energy wasting
QFM: Quadriceps femoris muscles
t0: time point before starting the interventions
t1: time point after 12 weeks
VAS: Visual Analog Scale
VR: Virtual reality
